# In Vivo Anti-Inflammatory, Analgesic, Sedative, Muscle Relaxant Activities and Molecular Docking Analysis of Phytochemicals from *Euphorbia pulcherrima*

**DOI:** 10.1155/2022/7495867

**Published:** 2022-04-13

**Authors:** Abdullah S. M. Aljohani, Fahad A. Alhumaydhi, Abdur Rauf, Essam M. Hamad, Umer Rashid

**Affiliations:** ^1^Department of Veterinary Medicine, College of Agriculture and Veterinary Medicine, Qassim University, Buraydah, Saudi Arabia; ^2^Department of Medical Laboratories, College of Applied Medical Sciences, Qassim University, Buraydah, Saudi Arabia; ^3^Department of Chemistry, University of Swabi, Anbar, Anbar, Khyber Pakhtunkhwa, Pakistan; ^4^Department of Food Science & Human Nutrition, College of Agriculture and Veterinary Medicine, Qassim University, Buraydah, Saudi Arabia; ^5^Department of Chemistry, COMSATS University Islamabad, Abbottabad Campus, Abbottabad 22060, Pakistan

## Abstract

*Euphorbia pulcherrima* is an important medicinal plant that is used in a traditional system for its curative properties such as analgesic potency, antipyretic, anti-inflammatory, sedation potential, and antidepressant and cure of diseases such as skin diseases. This study deals with the isolation of two flavonoids namely spinacetin (1) and patuletin (2) from chloroform fraction of *Euphorbia pulcherrima.* The isolated compound spinacetin (1) and patuletin (2) were screened for *in vivo* anti-inflammatory, analgesic, sedative, and muscle relaxant effects. Compounds 1 and 2 were assessed against hot plate-induced noxious stimuli at various doses which showed excellent (*p* < 0.05) analgesic effect in a dose-dependent manner. The muscle relaxant activity was determined by traction and inclined screening model, both compounds showed significant muscle relaxant activity with time. The sedative potential of isolated compounds 1 and 2 was determined by the open field model, both compounds showed good sedation (*p* < 0.05) at 20 mg/kg. The anti-inflammatory potential of compound 1 was recorded by histamine-induced paw edema and carrageen paw edema model, and in both models, compounds 1 and 2 showed strong effect at 20 mg/kg. Binding orientations, binding energy values, and computed inhibition constants (Ki) values revealed that the studied compounds have a good to excellent inhibition potential against *μ*-opioid receptors and COX-2.

## 1. Introduction

Medicinal plants are a key source of various classes of secondary metabolites [1]. The medicinal importance of plants is correlated due to the presence of natural products. There are several bioactive natural products isolated and are major constituents of the modern medicinal system [1]. Many plants are investigated for new drug discovery because the drugs prepared from plants are more effective and have no toxicity. Many medicinal plants have several biomedical applications such as Alzheimer's diseases [2], neurodegenerative [3], depressive [3], and metabolic disorders [4] as well as promote health and prevent disease [5].

*Euphorbia pulcherrima* is a member of the family Euphorbiaceae. Euphorbiaceae is also known as spurge or Euphorbias family. Euphorbiaceae is the largest flowering family among the Anthophyta. This family comprises 5000 species and 300 genera throughout the globe. All members of this family are a key and rich source of bioactive natural products. Members of this family are distributed in Saudi Arabia, Pakistan, and India. Different species of this genus is famous for its folk usage for the cure of various ailments. These species contain latex which is a rich source of bioactive secondary metabolites [6]. This genus comprises diterpenoids and other compounds which his documented for excellent anticancer and other properties [7]. For example, the essential oil isolated from *E. hirta* is used for the treatment of asthma and is also used as a mosquito repellent. Various phytochemicals including phytosterols, triterpenes, tannins, flavonoids, and polyphenols have been isolated from various parts of *E. hirta* [8]. Dry roots of *E. kansui* herbal remedy are used for the cure of ascites, edema, and cancer [8]. Various parts of dhudi (*E. hirta*) also comprise saponins, alkaloids, and flavonoids. *E. hirta* is employed for curing several ailments such as kidney stones, bronchial ailments, gastrointestinal problems, respiratory disease, and diabetes [9].

*Euphorbia pulcherrima* is also known as poinsettia or Christmas flower and is distributed on the Pacific coast of America as well as Mexico and Guatemala. *E. pulcherrima* is also found in various countries of Asia and Nepal [10]. The aerial part of *Euphorbia pulcherrima* is used in the traditional system for the treatment of ailments such as normal/slow transit constipation, skin disease, and rise of milk secretion in nursing mothers. Also, *E. pulcherrima* documented for various in vitro and in vivo biological activities [11]. Some species of Euphorbiaceae has been documented for numerous pharmacological potential such as central nervous system, inflammation, fever, neuropharmacological, and hypnotic [12]. Flavonoids isolated from *Euphorbia pulcherrima* have reported moulting properties with *Spodoptera littoralis* [13]. Flavonoids documented from other plants have been reported for anti-inflammation, analgesic, phosphodiesterase, and antidiarrheal activities [14–16].

Based on extensive pharmacological applications on the family Euphorbiacea, there looks to be unsatisfactory information on *E. pulcherrima*. The current finding aimed at the isolation of flavonoids namely spinacetin (1) and patuletin (2) and their in vivo pharmacological potential.

## 2. Materials and Methods

### 2.1. Collection of Plant

*Euphorbia pulcherrima* plant materials were collected in July 2015 from the botanical garden of the University of Peshawar, Peshawar Khyber Pakhtunkhwa, Pakistan. The collected plant sample was recognized by Prof. Dr. Barkatullah, Botany Department, Peshawar University, Khyber Pakhtunkhwa. The voucher specimen number (PUP545)/UOP was retained at the herbarium of the mentioned department.

### 2.2. Extraction and Isolation

The collected plant material was dried in shade, converted to powder, and then assessed to cold extraction with a polar solvent (methanol) for 20 days as per the reported method [17]. The extract obtained was filtered and then concentrated at reduced pressure and low through the rota evaporator. The crude extract 189 g was subjected to fractionation with various polarity solvents such as n-hexane, chloroform, ethyl acetate, and butanol. All fractions were subjected to think layer chromatography among which chloroform contain a maximum number of phytochemicals which was subjected for chromatographic analysis. Among chloroform fraction, 24.27 gm has exposed to column chromatographic analysis on silica gel and then eluted with a mixture of hexane and EtOAc (100 : 00 to 50 : 50). The column chromatography afforded 243 subfractions (AF1-TO AF243). The subfraction was combined based on TLC profile which yields ten subfactions (AFF1-AFF10). Among which AFF7 was subjected to repeated column chromatography on silica gel, eluting with hexane and EtOAc (40 : 60) which afforded compound 1 (1.62 g) and compound 2 (1.73 g). The chemical structure of isolated constituents 1 and 2 was identified by advanced spectroscopic analysis and comparing the spectral and physical data with reported data [18–20].

### 2.3. Animals

In vivo biological screening of compounds was performed by using Balb/c mice of both sexes having weight (24–27 g). The animals were housed in standard laboratory conditions, providing *ad libitum* water and food. The biological test was performed at the Department of Veterinary Medicine, College of Agriculture and Veterinary Medicine, Qassim University, Buraydah, Saudi Arabia, and University of Swabi, Pakistan, according to standard methods. The in vivo test involving animals was approved by local ethical committees (21-16-04, Committee of Research Ethics, Deanship of Scientific Research, Qassim University) and (UOS/Pharm-650, Department of Pharmacy, University of Swabi, KPK, Pakistan).

### 2.4. Anti-Inflammatory Screening

The isolated flavonoids spinacetin (1) and patuletin (2) were assessed for anti-inflammatory screening with carrageen paw edema and histamine-induced edema procedure as per the published procedure [21]. The mice were divided into various groups each group comprise six animals. The distributed groups of animals consist of negative control (10 mL/saline) and positive control (diclofenac; loratadine; 5 mg/kg). Tested groups of divided animals were treated with isolated flavonoids spinacetin (1) and patuletin (2) (5, 10, 15, and 20 mg/kg). Exactly after 30 minutes of the intraperitoneal administration, 1% of carrageen (0.05 mL) and histamine (0.1 mL) were transdermally administered in the right paw of each mice. The inflammation of PW was observed for each tested mice for six hours after injection of carrageen and histamine with the help of a plethysmometer. The anti-inflammatory of both tested flavonoids (1 and 2) was calculated with the help of the following formula:

(1) $$\begin{array}{c} \%\text{ effect}=\left(\frac{A-B}{A}\right)\times 100, \end{array}$$

where *A* is the paw edema of the control and *B* is the paw edema of tested flavonoids.

### 2.5. Analgesic Activity

The isolated flavonoids spinacetin (1) and patuletin (2) were also subjected to analgesic screening according to our published procedure [21]. In this model, all animals were distributed to various groups, and each group consists of six mice. Among the entire groups, only one group of mice was administered with normal saline (10 mL/kg) as a control for statistical calculation, and one group was administered with tramadol (5 mg/kg). The remaining groups of animals were treated with isolated flavonoids spinacetin (1) and patuletin (2) (5, 10, 15, and 20 mg/kg). The analgesic effect of tested compounds was identified through a hot-plate analgesiometer according to the reported method [21]. After 30 minutes of sample administration, every group of mice was screened for analgesic potential through hot plate and the exact time of the latency was observed in seconds. The percentage analgesic effect was identified as per the standard method [22].

### 2.6. Sedative Activity

The isolated flavonoids spinacetin (1) and patuletin (2) were also screened for sedation properties [23]. All animals were divided into various groups and each group consists of 6 animals. Among the entire group, the control was administered with 0.5 mg/kg of diazepam. The sedative potential of flavonoids spinacetin (1) and patuletin (2) was performed as per the reported method. According to this procedure, after 30 minutes of administration, every group of animals should be placed in a special design box containing lines, the line crossed by every mouse observed. Amount tested groups of animals, that group of animals with the delayed movement was considered as a sedative while the rest of groups of mice which crossed more lines were considered none sedative.

### 2.7. Muscle Relaxant Screening

#### 2.7.1. Inclined Plane Method

The isolated flavonoids spinacetin (1) and patuletin (2) were also screening for muscle relaxant potential in an include plane procedure as per the published method [23]. Include plane model composed 2 plywood boards linked to each other among which one side of the plywood board shaped the base and the other side of the plywood board was connected to the base at almost 65 degrees. All animals were divided into various groups, and each group consists of 6 animals. Animals of a different group in this screening were treated with standard (diazepam; 1 mg/kg), normal saline (10 mL/kg), and isolated flavonoids spinacetin (1) and patuletin (2) (5, 10, 15, and 20 mg/kg). After treatment of all samples including spinacetin (1) and patuletin (2), standard, and saline with various time intervals (30, 60, and 90 minutes), the animals was observed to fall or not for around 30 seconds on putting on the upper part of an inclined plane.

#### 2.7.2. Traction Method

The isolated flavonoids spinacetin (1) and patuletin (2) were also screened for muscle relaxant potential by using the traction model [23]. This model comprises a metal wire cover with rubber, where ends of this metal wire were tightly stretched and reinforced with stands and were kept about 60 to 70 cm above the bench. To screen all samples, the animals were distributed into fourteen groups and each group comprises 5 animals. Among the groups, one group received distilled water, one received diazepam which was considered as a negative and positive group. The remaining groups of animals were administered with spinacetin (1) and patuletin (2) in various doses such as 5, 10, 15, and 20 mg/kg i.p. The muscle relaxation properties were observed for all groups of animals at various time intervals, i.e., 30, 60, and 90 minutes. Every mouse in this assay was suspended on the metal wire from hind legs, and the floppy time was recorded for nearly 5 seconds. This failure of mice to hang in the metal wire in less than 5 sec indicated the presence of muscle relaxant property for spinacetin (1) and patuletin (2).

### 2.8. Toxicological Profile

The purified spinacetin (1) and patuletin (2) was screened for toxicological study as per reported method [23]. All animals were distributed into respective groups, each group comprising six animals. The purified spinacetin (1) and patuletin (2) were administered to various groups of animals at doses 10, 20, 100, and 200 mg/kg. The tested animals were kept under observation for 2 days. The mortality was calculated from the number of dead and surviving animals of each group.

### 2.9. Statistical Analysis

The results of anti-inflammatory, analgesic, sedative, and muscle relaxant screening are represented as the mean ± standard error of the mean (SEM) to identify the significant difference in all tested animals. The results achieved were evaluated to one-way analysis of variance (ANOVA). The analysis was done with help of Dunnett's multiple assessment screening using GraphPad Prism 5.

## 3. Molecular Docking

The isolated spinacetin (1) and patuletin (2) were docked in the binding site of COX-2 and opioid receptors by using Molecular Operating Environment (MOE) software (201.2016.0802) and AutoDock (*v* 4.0) [22, 24]. Online database of biological macromolecules RCSB PDB was used to download three-dimensional (3D) crystal structures of selected proteins. The accession codes of these proteins are 1CX2 for COX-2, 4COF for *μ*-opioid receptor, and 5C1M for GABA_A_ receptor.

Next, we performed validation of docking protocol by using the redock method. All the native ligands were extracted and docked into the binding site of their respective proteins. A comparison between redocked conformation and experimental confirmation showed a root mean square deviation with a limit (<2.0 Å).

2D structures of isolated compounds were drawn in ChemDraw and then exported to the MOE and AutoDock. The structures were saved in a 3D format in MOL2 (for AutoDock) and molecular database (.mdb) format for MOE, and saved as 3D structures. Preparation of downloaded proteins was performed by using our previously reported methods [25]. For each compound, we set the default parameters for docking simulations. Finally, 3D and 2D analysis of each docked pose was performed by using MOE and a discovery studio visualizer [25].

## 4. Results

### 4.1. Characterization of Spinacetin (1) and Patuletin (2)

**Figure 1:**
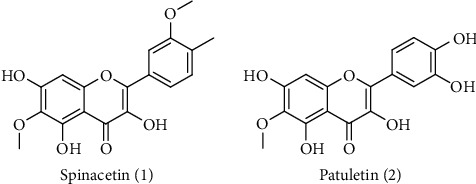
Chemical structures of spinacetin (1), patuletin (2) isolated from *E. pulcherrima*.

Spinacetin (1) and patuletin (2) were purified from chloroform soluble fraction of *E. pulcherrima* through chromatographic analysis. The structure of spinacetin (1) and patuletin (2) (Figure 1) was characterized by advanced nuclear magnetic resonance spectroscopy and mass spectrometry.

### 4.2. Anti-Inflammatory Effect

**Figure 2:**
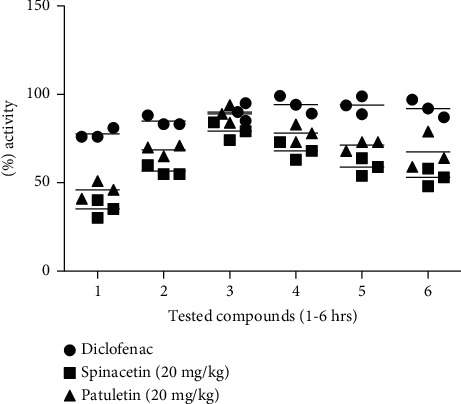
Anti-inflammatory effect of spinacetin (1) and patuletin (2; 20 mg/kg) of the *E. pulcherrima* on carrageenan paw in mice. The obtained results are shown as ±SEM for six groups of animals. The analysis of data was induced paw edema in mice. The achieved results are shown as ±SEM for 6 groups of animals. The calculation was performed by ANOVA followed by Dunnett's test.

The results of spinacetin (1) and patuletin (2) showed that it exhibited 35.22 and 46.00% attenuation in carrageenan-induced paw edema model which reached a maximum of 79.22 and 89.01% after 3^rd^ hours at 20 mg/kg and remain good in the 5^th^ hours. Both tested compounds showed mild effect at 5, 10, and 15 mg/kg as compared to 20 mg/kg. Diclofenac sodium was used as a positive control in this investigation which showed outstanding effect as compared to spinacetin (1) and patuletin (2) (Figure 2).

**Figure 3:**
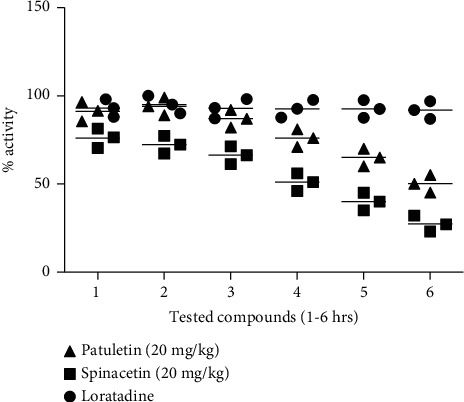
Anti-inflammatory effect of spinacetin (1) and patuletin (2; 20 mg/kg) of *E. pulcherrima* on histamine-induced paw edema in mice. The achieved results are shown as ±SEM for 6 groups of animals. The calculation was performed by ANOVA followed by Dunnett's test.

Likewise in the histamine paw edema model, spinacetin (1) and patuletin (2; 20 mg/kg) exhibited 76.45 and 91.45% activity for the initial phase (at the 1^st^ hrs) while 78.33 and 94.00% (after 2^nd^ hrs) and continues up to 3^rd^ hours. Spinacetin (1) and patuletin (2) exhibited excellent activity as compared to loratadine (Figure 3).

### 4.3. Analgesic Effect

Spinacetin (1) and patuletin (2) isolated from *E. pulcherrima* exhibited promising (*p* < 0.001) central analgesic potency at tested doses (5, 10, 15, and 20 mg/kg) as represented in Table 1. Spinacetin (1) and patuletin (2) increase the latency time from the start of treatment of compounds (1 and 2) and sustained a promising increase (*p* < 0.001) up to 120 minutes. Both tested compounds exhibited lower effects at low tested doses.

### 4.4. Sedative Effect

Sedative potentials of spinacetin (1) and patuletin (2) are given in Table 2. Spinacetin (1) and patuletin (2) showed promising (*p* < 0.001) sedation effect at tested doses (5, 10, 15, and 20 mg/kg) in dose-dependent manner. Patuletin (2) exhibited significant (*p* < 0.001) activity at 20 mg/kg, as represented by hindering the mobility of animals in a distinct box.

### 4.5. Muscle Relaxant Effect

Spinacetin (1) and patuletin (2) isolated from *E. pulcherrima* were also screened for muscle relaxant effect on inclined plan and traction model, which showed promising muscle relaxant potential (Table 3). Spinacetin (1) and patuletin (2) exhibited significant effects in the dose- and time-dependent manner. Both tested compounds possess good activity in both models. The effect of both compounds was excellent from the start of the experiment against the standard drug.

### 4.6. Toxicological Study

The purified spinacetin (1) and patuletin (2) was screened for toxicological study in the higher doses 10, 20, 100, and 200 mg/kg. Spinacetin (1) and patuletin (2) was administered to the animals intraperitoneally (i.p). The tested animals were kept for 2 days under observation. The number of surviving and dead animals was recorded. All groups of treated animals survived all tested doses. Interestingly, spinacetin (1) and patuletin (2) was found safe at all tested doses.

### 4.7. Docking Studies on *μ*-Opioid and GABA_A_ Receptors

We have performed the docking simulations on GABAA receptors as well as on *μ*-opioid receptors. The accession codes for the downloaded proteins from the protein data bank are 5C1M for *μ*-opioid and 4COF for GABA receptors. After the validation of the docking protocols, we docked spinacetin (1) and patuletin (2) into the binding sites of the downloaded and prepared targets.

**Figure 4:**
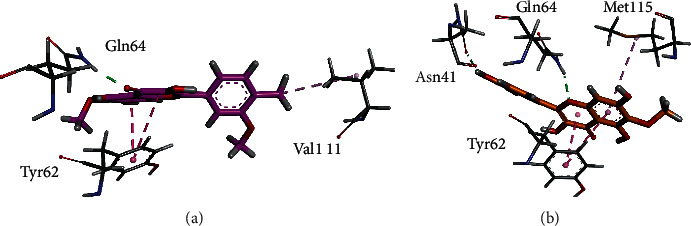
The 3D interaction plots of spinacetin (1) and patuletin (2) into the binding site of GABA (PDB Id: 4COF). (a) Spinacetin (pink) and (b) patuletin (orange).

Three-dimensional (3D) interaction plots of isolated compounds show that spinacetin (1) forms a hydrogen bond interaction with Gln64. The phenyl ring of the compound forms *π*-*π* interactions with Tyr62. His54 forms with the phenyl ring (Figure 4(a)), while patuletin (2) forms two hydrogen bond interactions and one *p*-*π* interaction. Asn41 and Gln64 forms hydrogen bond interactions, and Tyr62 forms *p*-*π* interactions (Figure 4(b)).

**Figure 5:**
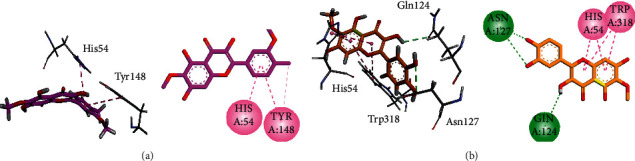
The 3D and 2D interaction plots of spinacetin (1) and patuletin (2) into the binding site of *μ*-opioid (5C1M). (a) Spinacetin (pink) and (b) patuletin (orange).

Three-dimensional (3D) and two-dimensional (2D) interaction plots of the isolated compounds in the binding site of *μ*-opioid (5C1M) are shown in Figure 5. The phenyl ring of spinacetin (1) established two *p*-*π* interactions with His54 and Tyr148 (Figure 5(a)). Patuletin (2) forms hydrogen bond interactions with Gln124 and Asn127, while chromen-4-one ring forms bifurcated *π*-*π* interactions with His54 and Trp318 (Figure 5(b)).

**Figure 6:**
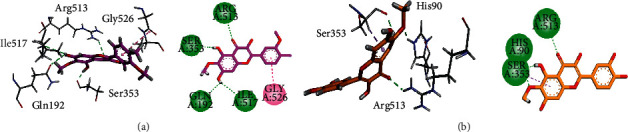
The 3D and 2D interaction plots of isolated compounds into the binding site of COX-2 (PDB Id: 1CX2). (a) Spinacetin (pink) and (b) patuletin (orange).

We docked isolated compounds into the binding site of COX-2 isoform (PDB Id: 1CX2). 3D/2D interaction plots of the studied compounds are shown in Figure 6. It can be observed from these interaction plots that both compounds interact with the amino acid residues (His90, Gln192, Ser353, and Arg531) present in the selectivity pocket of the COX-2. The hydroxyl groups of compounds 1 and 2 form hydrogen bond interactions with selectivity pocket residues His90, Ser353, and Arg513. Ile517 also establishes a hydrogen bond with the hydroxyl group of compound 1 (Figures 6(a) and 6(b)).

Furthermore, we performed the docking studies by using the AutoDock 4.0 software. The purpose is to compute the inhibition constant (Ki) of the isolated compounds against the tested targets. The binding energy values computed via MOE and AutoDock and Ki values computed via AutoDock are listed in Tables 4-5.

## 5. Discussion

*E. pulcherrima* is employed for multimedicinal usage for the central analgesic potency, antipyretic, anti-inflammatory, sedation potential, and antidepressant and for the cure of skin diseases. Due to the diverse biological potential, the isolation and structure determination of secondary metabolites followed by in vivo biological screening is important to identify the secondary metabolites responsible for the diverse biological application. In this finding, compounds 1 and 2 were isolated and screened for analgesia, muscle relaxation, sedative, and anti-inflammatory properties.

Prostaglandin is an important product of arachidonic acid by cyclooxygenase enzyme. Majority of the cyclooxygenase enzyme inhibitors are recognized as active painkillers, also as anti-inflammatory agents [26, 27]. It is a big challenge for a researcher to discover new potent and less or nontoxic inhibitors. Spinacetin (1) and patuletin (2) also attenuated inflammation in both tested models, i.e., carrageenan and histamine. Carrageenan paws edema model consists of two phases: the initial phase edema is recognized to the local release of histamine serotonin and bradykinins while the further phase is due to overproduction of PG [27]. In the tested model, spinacetin (1) and patuletin (2) exhibited significant analgesic activity against the standard drug.

The muscle relaxant effect of spinacetin (1) and patuletin (2) in both models that is inclined plan and traction model was analysed as per well-established methods. Spinacetin (1) and patuletin (2) exhibited a strong muscle relaxant effect. In both tested models, the relaxation of muscle was assessed after 30, 60, and 90 minutes of treatment of spinacetin (1) and patuletin (2). An outstanding muscle relaxant effect was observed after 60 minutes of spinacetin (1) and patuletin (2) administration at a higher dose.

Similarly, spinacetin (1) and patuletin (2) were also evaluated for their sedative properties which showed excellent activity. The sedative activity of these secondary metabolites has been recognized with their antihistaminic potential [28]. The antihistaminic potential may be similar to H1 receptor blocker, i.e., pheniramine which has an excellent sedative potential; thus, our screen compounds (1 and 2) have outstanding sedative potential. From our outstanding results, we recommend researchers for comprehensive and detailed screening as leading molecules for curing various diseases. The excellent anti-inflammatory, analgesic, sedative, and muscle relaxant potential of spinacetin (1) and patuletin (2) provide a strong scientific background for folkloric use of *E. pulcherrima* for treatment of various diseases.

To further support the in vitro result findings, we performed molecular docking analysis on the selected target receptors. First of all, docking simulations were performed on GABAnergic and *μ*-opioid targets. Both isolated compounds showed interactions with the amino acid residues of the studied targets. *μ*-Opioid receptor (*μ*OR) and membrane proteins are considered as key therapeutic targets for pain. The binding of ligands to the target identifies the responses which are resulted into the activation of signaling pathway. A number of crystal structures have been solved for *μ*OR that enable the drug discovery scientists to design a new ligand. Insights into the binding pattern of native agonist BU72 (PDB 5C1M) revealed that it interacts with His54, Asp147, and Tyr148 *via* hydrogen bond interactions. Here, in our study, the phenyl ring of spinacetin (1) established *π*-*π* interactions with His54 and Tyr148, while the chromen-4-one ring of patuletin (2) forms bifurcated *π*-*π* interactions with His54 and Trp318.

The computed binding energy values by using MOE and AutoDock revealed that both compound bind to GABAa receptor with almost same affinity. However, compound 1 showed slight more affinity when bound with *μ*OR. We moved further and computed the inhibition constant (Ki) of these compounds. Hence, we used AutoDock to calculate the Ki values of the compounds. Both compounds showed good inhibition constant against *μ*OR. The Ki values are in low micromolar range (Table 4), while for GABA receptors, the compounds exhibited moderate Ki values.

In the next step, we studied the inflammation pathway *via* COX-2 inhibition. Carrageenan-induced paw edema model is considered as COX-2-dependent model of inflammation. Spinacetin (1) exhibited excellent Ki value in submicromolar concentration (Ki = 0.631 *μ*M). Binding pose revealed that the isolated compound is more oriented towards the additional or the selectivity pocket of COX-2 and established significant hydrogen bond interactions with the key amino acid residues (His90, Gln192, and Arg513).

## 6. Conclusions

It is concluded that spinacetin (1) and patuletin (2) isolated from *E. pulcherrima* showed excellent anti-inflammatory, analgesic, sedative, and muscle relaxant effects. Thus, our current research provides a scientific rationale to the traditional use of *E. pulcherrima* for the cure of various diseases. Furthermore, spinacetin (1) and patuletin (2) are outdating candidates for detailed research study to find its clinical application. Compounds 1 and 2 showed excellent (*p* < 0.05) analgesic effect in a dose-dependent manner. The muscle relaxant activity results showed significant muscle relaxant activity with time, while sedative potential of compounds under study showed good sedation (*p* < 0.05) at 20 mg/kg. Moreover, the anti-inflammatory potential of compounds determined *via* histamine-induced paw edema and carrageen paw edema model showed strong effect at 20 mg/kg. Inhibition constants (Ki) values computed *via* the AutoDock software revealed that these compounds have a good to excellent inhibition potential against GABA and COX-2, while binding orientation and interaction plots also showed binding affinities the studied targets. The excellent anti-inflammatory, analgesic, sedative, and muscle relaxant potential of spinacetin (1) and patuletin provides a strong scientific background for folkloric use of *E. pulcherrima* for treatment of various diseases.

## Figures and Tables

**Table 1:** Analgesic effect of compounds (1 and 2) isolated from *E. pulcherrima*.

Group	Dose (mg/kg)	Time in minutes			
		30	60	90	120
Normal saline	10 mL	9.25 ± 0.07	9.24 ± 0.08	9.25 ± 0.15	9.23 ± 0.11
Tramadol	10	25.00 ± 0.08^*∗∗∗*^	26.28 ± 0.07^*∗∗∗*^	25.50 ± 0.06^*∗∗∗*^	25.70 ± 0.11^*∗∗∗*^
Compound 1	5	11.55 ± 0.30	12.90 ± 0.43	12.80 ± 0.35	12.77 ± 0.40
	10	14.98 ± 0.98	15.55 ± 0.21	15.30 ± 0.45	15.20 ± 0.46
	15	17.32 ± 0.40	18.90 ± 0.33	18.30 ± 0.43	18.20 ± 0.49
	20	20.00 ± 0.33	21.03 ± 0.23	20.88 ± 0.32	20.80 ± 0.55
Compound 2	5	13.66 ± 0.10	14.96 ± 0.18	14.80 ± 0.20	14.60 ± 0.45
	10	16.90 ± 0.39	17.55 ± 0.23	17.36 ± 0.43	17.31 ± 0.55
	15	19.80 ± 0.21	20.93 ± 0.27	20.88 ± 0.40	20.59 ± 0.40
	20	22.98 ± 0.60	23.84 ± 0.73	23.70 ± 0.80	23.65 ± 0.90

The recorded results are represented as the mean ± SEM of all animals' tolerance to thermal stimuli in seconds.

**Table 2:** Effect of compounds (1 and 2) isolated from *E. pulcherrima* (locomotive activity).

Sample	Dose (mg/kg)	No. of lines crossed
Control	5 mL	129.35 ± 3.24
Diazepam	0.5	9.32 ± 0.45^*∗∗∗*^
Compound 1	5	70.54 ± 1.01
	10	65.87088
	15	54.76 ± 0.66^*∗∗*^
	20	44.87 ± 0.98^*∗∗*^
Compound 2	5	66.21 ± 1.06
	10	57.91 ± 1.00^*∗∗*^
	15	46.98 ± 0.97^*∗∗*^
	20	37.66 ± 0.93^*∗∗∗*^

The recorded results are represented as the mean ± SEM of all animals' tolerance to thermal stimuli in seconds.

**Table 3:** Muscle relaxant effect of compounds (1 and 2) isolated from *E. pulcherrima*.

Group	Dose (mg/kg)	Inclined plane test (% effect)			Traction test (effect)		
		30 mints	60 mints	90 mints	30 mints	60 mints	90 mints
Distilled water	10 mL	0.00 ± 0	0.00 ± 0	0.00 ± 0	0.00 ± 0	0.00 ± 0	0.00 ± 0
Diazepam	1	100 ± 0.00	100 ± 0.00	100 ± 0.00	100 ± 0.00	100 ± 0.00	100 ± 0.00
Compound 1	5	25.32 ± 1.44	29.90 ± 1.37	31.66 ± 1.20	26.61 ± 1.32	27.22 ± 1.00	26.09 ± 1.33
	10	30.22 ± 1.40	34.65 ± 1.33	36.98 ± 1.34	31.88 ± 1.34	32.67 ± 1.03	31.07 ± 1.20
	15	40.23 ± 1.35	47.09 ± 1.32	48.67 ± 1.18	41.34 ± 1.09	42.34 ± 1.04	41.22 ± 1.18
	20	46.09 ± 1.36	52.00 ± 1.28	53.99 ± 1.23	47.32 ± 1.05	48.23 ± 1.06	47.23 ± 1.23
Compound 2	5	30.21 ± 1.23	36.87 ± 1.45	37.32 ± 1.02	31.11 ± 1.09	32.034 ± 1.00	31.98 ± 1.32
	10	36.09 ± 1.67	43.34 ± 1.32	45.23 ± 1.07	37.23 ± 1.23	38.012 ± 1.34	37.56 ± 1.07
	15	47.03 ± 1.40	55.13 ± 1.22	57.45 ± 1.44	48.87 ± 1.09	49.03 ± 1.09	47.66 ± 1.23
	20	55.08 ± 1.33	63.11 ± 1.00	64.98 ± 1.76	56.32 ± 1.23	58.02 ± 1.23	56.76 ± 1.02

The recorded results are represented as the mean ± SEM of all animals' tolerance to thermal stimuli in seconds.

**Table 4:** Binding energy and inhibition constant of the spinacetin (1) and patuletin (2) calculated by AutoDock into the binding site of *μ*-opioid and GABA_A_ receptors.

Compound no.	GABAA receptor (PDB Id: 4COF)			*μ*-opioid receptor (PDB Id: 5C1M)		
	MOE	AutoDock		MOE	AutoDock	
	BE (kcal/mol)	BE (kcal/mol)	Ki (*μ*M)	BE (kcal/mol)	BE (kcal/mol)	Ki (*μ*M)
1	−5.8221	−6.49	17.44	−7.1978	−7.89	4.84
2	−5.8935	−6.33	22.82	−6.7992	−7.11	6.19

**Table 5:** Binding energy and inhibition constant of the isolated spinacetin (1) and patuletin (2) calculated by AutoDock into the binding site of COX-2 receptors.

Compound no.	COX-2 (PDB Id: 1CX2)		
	MOE	AutoDock	
	BE (kcal/mol)	BE (kcal/mol)	Ki (*μ*M)
1	−7.7702	−8.46	0.631
2	−6.8761	−7.86	1.74

## Data Availability

The spectroscopic data of isolated compounds (1–3) are available on request to the contact authors. The graphical abstract is given in supplementary data.

## Supplementary Materials

Supplementary Materials Graphical abstract illustrating the in vivo anti-inflammatory, analgesic, sedative, and muscle relaxant activities and molecular docking analysis of phytochemicals from *Euphorbia pulcherrima*.
