# Supplementary material for: In Vivo Anti-Inflammatory, Analgesic, Sedative, Muscle Relaxant Activities and Molecular Docking Analysis of Phytochemicals from Euphorbia pulcherrima
Source: Evid Based Complement Alternat Med. 2022 Apr 13;2022:7495867. doi: 10.1155/2022/7495867 (PMC9205722; doi:10.1155/2022/7495867)
Supplement: Supplementary file 1 [file 7495867.f1.docx]

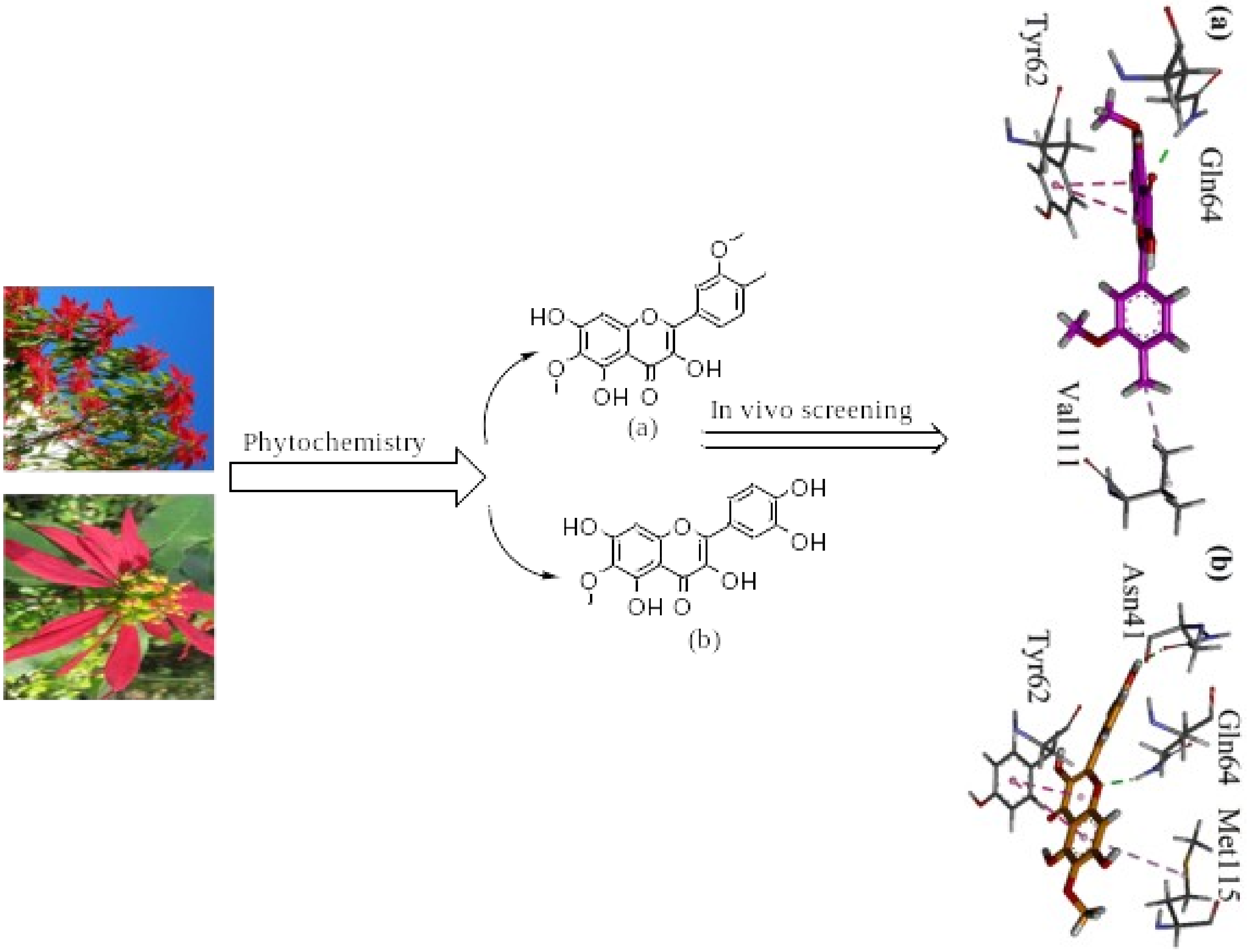

Phytochemistry
HO
OH
OH
O
(a)
In vivo screening
OH
(b)
Gln64
Tyr62
Val111
Asn41
Met115
